# First Molecular and Phylogenetic Characterization of Equine Herpesvirus-1 (EHV-1) and Equine Herpesvirus-4 (EHV-4) in Morocco

**DOI:** 10.3390/ani15010102

**Published:** 2025-01-05

**Authors:** Zineb El Brini, Ann Cullinane, Marie Garvey, Ouafaa Fassi Fihri, Siham Fellahi, Farid Amraoui, Chafiqa Loutfi, Ghizlane Sebbar, Romain Paillot, Mohammed Piro

**Affiliations:** 1 Department of Medicine, Surgery, and Reproduction, Agronomy and Veterinary Institute Hassan II, Rabat 10000, Morocco; vetpiro@yahoo.fr; 2 Virology Unit, The Irish Equine Centre, Johnstown, Naas, Co., W91 RH93 Kildare, Ireland; acullinane@irishequinecentre.ie (A.C.); mgarvey@irishequinecentre.ie (M.G.); 3 Department of Microbiology, Immunology and Contagious Diseases, Agronomy and Veterinary Institute Hassan II, Rabat 10000, Morocco; fassifihri.ouafaa@gmail.com; 4 Department of Veterinary Pathology and Public Health, Agronomy and Veterinary Institute Hassan II, Rabat 10000, Morocco; fellahisiham2015@gmail.com; 5 Society of Veterinary Pharmaceutical and Biological Productions (Biopharma), Rabat 10000, Morocco; amraoui.farid@gmail.com (F.A.); c.loutfi@biopharma.ma (C.L.); sghizou@gmail.com (G.S.); 6 Writtle School of Agriculture, Animal and Environmental Science, Faculty of Science and Engineering, Anglia Ruskin University, Lordship Road, Writtle, Chelmsford CM1 3RR, UK; romain.paillot@aru.ac.uk

**Keywords:** equine herpesvirus 1, equine herpesvirus 4, equine herpesvirus 8, multiplex PCR, isolation, molecular characterization, Morocco

## Abstract

This study aimed to investigate the molecular prevalence and genetic characterization of EHV-1 and EHV-4 in equid populations in Morocco. A total of 154 equids (114 horses, 9 donkeys, and 31 mules) were sampled, with nasal swabs and tissue samples subjected to multiplex real-time PCR for the detection of EHV-1 and EHV-4. Additionally, an isolate from the tissue of an aborted horse fetus was included in the analysis. A subset of EHV-positive samples underwent virus isolation followed by whole-genome sequencing. PCR assays revealed that 42 samples (27%) tested positive for EHV-4, while only 3 samples (1.94%) were positive for EHV-1. Attempts to isolate the virus from EHV-4-positive samples were unsuccessful. However, virus isolation was successful in an EHV-1-positive nasopharyngeal sample from a donkey. Phylogenetic and molecular characterization reclassified the EHV-1 isolated from the donkey as an EHV-8. Meanwhile, the EHV-1 isolated from the aborted fetal tissue was classified as a clade 1 EHV-1 virus. This study is the first to report the molecular prevalence and genetic characterization of EHV-1 and EHV-4 in equid populations in Morocco, providing valuable insights into the distribution and genetic diversity of these viruses in the region.

## Simple Summary

This study evaluated the molecular prevalence and genetic characterization of equine EHV-1/4 in equids populations in Morocco. Among 154 samples analyzed, 27% tested positive for EHV-4, while only 1.94% were positive for EHV-1. Virus isolation was unsuccessful in EHV-4-positive samples but EHV-1 was isolated from a positive donkey sample. Phylogenetic and molecular analyses reclassified the EHV-1 isolate from the donkey as EHV-8, while the EHV-1 isolate from aborted fetal tissue was identified as clade 1 EHV-1. This study confirms the active circulation of EHV-1 and EHV-4 in Moroccan equines, reports the first detection of EHV-8 in a donkey in Africa, and highlights EHV-4′s prevalence and its association with respiratory disorders, abortions, and neonatal deaths.

## 1. Introduction

Equine herpesvirus-1 (EHV-1) and equine herpesvirus-4 (EHV-4), recently designated as Varicellovirus equidalpha 1 and 4 [1], present considerable economic challenges to the global equine industry [2]. Equine Rhinopneumonitis is the collective term for clinical disease entities of equids that may occur as a result of infection by either EHV1 or EHV4. It is usually estimated that 80–90% of horses encounter the virus by the age of two [3]. Initial infection occurs through inhalation of aerosols or oral–nasal contact with contaminated fomites. Once in the respiratory tract, EHV-1 and EHV-4 rapidly proliferate in epithelial cells of the upper respiratory tract [3,4,5]. Subsequent to the initial inflammatory response and cellular necrosis, the airway epithelium erodes, leading to the shedding of the virus in respiratory secretions [3,6].

The clinical outcome of infected individuals is influenced by various factors, including age, physical condition, immune status, infectious agent dosage, and whether it is a primary infection, reinfection, or reactivation from a latent state [7]. Unlike EHV-1, EHV-4 infections typically remain confined to the upper respiratory tract. Leukocyte viremia is exceptionally rare and not consistently associated with EHV-4. As a result, EHV-4 is recognized as the predominant viral cause of equine acute respiratory disease [8,9,10,11].. Additionally, EHV-4 is sporadically linked to abortion and, on rare occasions, neurological disorders [12].

Furthermore, cellular viremia during an EHV-1 infection allows the virus to disseminate within infected leukocytes, leading to infection of endothelial cells of inner organs and subsequent multifocal vasculitis in affected blood vessels [5,13]. Consequently, the disease manifests itself in secondary forms, such as abortion, neonatal death, chorioretinopathy, and encephalomyelitis [8,9,10]. Both viruses establish latent infections in the sensory ganglia [14,15] and lymphoid tissues of their hosts [16]. Therefore, infected hosts will become latently infected and will henceforth be lifelong carriers of these viruses. The reactivation of EHV-1 or EHV-4 can occur during periods of stress, and this recrudescent virus is then shed from the respiratory tract to infect other susceptible horses [17]. Several techniques are used to diagnose EHV-1/4 infections, including the “gold standard” virus isolation method, serological tests, and nucleic acid detection techniques (PCR). However, virus isolation is a time-consuming method, and serological techniques only indicate that the animal has been exposed to the virus by natural infection or by vaccination; acute and convalescent paired serum samples with significant increase in antibody titer (four-fold) are required to indicate if the exposure was recent [8,18,19]. Consequently, the preferred diagnostic method is PCR, specifically real-time PCR (qPCR), owing to its rapidity, high sensitivity, specificity, and cost-effectiveness [20,21]. This technique provides a very useful diagnostic tool for the studies of infectious disease and is a valuable aid for screening large numbers of samples [11,22,23,24,25,26,27].

The complete nucleotide sequences of the genomic DNA of both EHV-1, EHV-4 and their close relative, EHV-8, led to the classification of the virus in the genus *Varicellovirus* (family *Herpesviridae*). Their genome consists of long and short unique regions (UL and US, respectively), the former flanked by a small inverted repeat (TRL/IRL) and the latter by a large inverted repeat (TRS/IRS) [28,29,30,31,32]. The genome contains 76 open reading frames (ORFs) predicted to encode functional proteins, 4 of which are duplicated in the TRS/IRS region. The average nucleotide composition of the EHV-1 genome is 56.7% G + C, although it is significantly higher in the TRS/IRS region, at 67% [30].

Despite the worldwide financial effect of these two alphaherpesviruses on the equine industry, no research has been performed in Morocco to emphasize the prevalence of these pathogens in respiratory disease, abortions, neonatal deaths or neurological damage. In contrast, neighboring countries have reported varying prevalence rates through molecular studies. Algeria [33] identified a molecular prevalence of 2% for EHV-1 and 14% for EHV-4, while Tunisia [34] reported a molecular prevalence of 3.69% for EHV-1 with no detection of EHV-4. However, efforts to sequence and characterize circulating herpesvirus strains remain absent in Morocco and the broader North African region.

The primary objective of this study is to molecularly detect EHV-1 and/or EHV-4 infections within the Moroccan equid population. Samples were collected from horses, donkeys, and mules presenting with clinical symptoms potentially associated with equine herpesvirus infection. Beyond detection, this study focuses on the molecular characterization of EHV-1 and EHV-4 strains circulating in Morocco, aiming to address a critical knowledge gap in the region’s understanding of these pathogens.

## 2. Materials and Methods

### 2.1. Molecular Prevalence

#### 2.1.1. Sample Collection

From 2016 to 2021, 154 equids, consisting of 114 horses, 9 donkeys, and 31 mules, were sampled across four regions in Morocco: Rabat, Marrakech-Tadla, Fez-Meknes, and Casablanca. Among the equids studied, 34 horses were primarily used for breeding, while the remaining horses, along with the donkeys and mules, served as draft animals. Tissue samples (liver, lung, spleen, and placenta) were collected from 24 aborted fetuses over 6 months of gestation or from neonatal deaths (under 15 days old). Additionally, 130 nasopharyngeal swabs were obtained from equids exhibiting respiratory symptoms (*n* = 124, such as nasal discharge, coughing, and fever) or neurological signs (*n* = 6, including ataxia with ascending paralysis). These swabs were immediately placed on ice in sterile transport medium containing minimum essential medium with 5% antibiotics (20,000 IU/mL penicillin, 10,000 µg/mL streptomycin, and 5000 µg/mL kanamycin). The samples were transported to the laboratory on ice packs within 24 h and processed within hours or stored at −80 °C until needed. The study also included an isolate from liver tissue of an aborted horse fetus collected from the Bouznika stud during the 2009–2010 foaling season.

#### 2.1.2. PCR

Real-time PCR assays for the initial diagnosis of EHV-1 and EHV-4 were performed with primers and probes targeting the highly conserved glycoprotein B (gB) gene which allow discrimination between EHV-1 and EHV-4 [35]. The probes used were TaqMan hydrolyzed probes (Applied Biosystems, Scoresby, Victoria, Australia)with a fluorescent reporter FAM for EHV-1 and VIC for EHV-4. This allowed the detection of both EHV-1 and EHV-4 in a single reaction mix, i.e., multiplex PCR, as described previously by Diallo et al. [24,36]. Extraction of DNA from collected samples and infected cell cultures (isolate) was performed using the MagMAX™ Core Nucleic acid purification kit (Thermo Fisher Scientific, Merelbeke, Belgium) according to the manufacturer’s instructions. PCR reaction was performed in a total volume of 25 µL containing 0.4 μM from each primer, 0.2 µM probe, 2 × PCR buffer, 2 mM MgSO4, Platinum taq mix and 5 μL from DNA according to the PCR protocol. All reagents were supplied by Invitrogen SuperScript™ III Platinum™ One-Step qRT-PCR (Thermo Fisher Scientific, Carlsbad, CA, USA). The DNA was amplified under the following conditions in a smart cycler (Cepheid, Sunnyvale, CA, USA): a primary denaturation step at 95 °C for 15 min, 40 cycles using the following settings: initial denaturation at 94 °C for 15 s, annealing and extension at 64 °C for 6 s. Positive and negative controls (NTC; NO Template Control, composed of the PCR reaction mix) were included in each run.

Reference strains Kentucky D for EHV-1 and 405/75 for EHV-4 were used as positive controls for the extraction and amplification of EHV-1 and EHV-4. These reference strains were provided by the Department of Veterinary Sciences at the University of Torino (Italy).

At the World Organisation for Animal Health (WOAH) reference laboratory for Equine Rhinopneumonitis located at the Irish Equine Centre, DNA was extracted using the MagVet Universal Isolation Kit (Life Technologies, Austin, Texas, USA) and the Kingfisher Flex Magnetic Particle Processor (Thermo Scientific/Life Technologies, Singapore) as per the manufacturer’s guidelines. Real-time PCR targeting glycoprotein C of EHV-1 and glycoprotein B of EHV-4 was performed on purified nucleic acids using primers and probes for EHV1 (EHV-1 gC Forward Primer: 5′-GCGGGCTCTGACAACACAA-3′; EHV-1 gC Reverse Primer: 5′-TTGTGGTTTCATGGGAGTGTGTA-3′; EHV-1 gC probe FAM-TAACGCAAACGGTACAGAA-MGB) and EHV4 (EHV-4 gB Forward Primer: 5′-TGTTGCCGTGCTATCTGTG-3′; EHV-4 gB Reverse Primer: 5′-GCTGGAGTACGGTGTATAGAA-3′; EHV-4 gB probe Cy5-TTATTGGCGAAGAGACTGTGGTA-BHQ) with the AgPath-ID One-Step RT-PCR (Applied Biosystems/Life Technologies, Austin, Texas, USA). After denaturation at 95 °C for 10 min, the amplification conditions encompassed 40 cycles of 95 °C for 15 s and 60 °C for 45 s. Amplification, data acquisition, and data analysis were carried out with the 7500 Real-Time PCR system (Applied Biosystems by Life Technologies, Singapore).

#### 2.1.3. Statistical Analysis

The age groups were based on biological criteria, taking into consideration the life expectancy of the horses (approximately 25–30 years) and the distribution of the horses in the study, aiming to include, as much as possible, a similar number of individuals within each category.

A chi-square (χ^2^) test was conducted to evaluate the associations between PCR-positive and PCR-negative results for EHV-1/4 and various variables (year, age, sex, species, and region). A *p*-value of less than 0.05 was considered statistically significant. Statistical analysis was performed using the JMP (ver. 17.0.0) software [37] software package.

### 2.2. Viral Culture and Sequencing

#### 2.2.1. Virus Isolation

For viral culture, only samples with a Ct (threshold cycle) value of less than 30, as determined by PCR results, were selected to ensure sufficient viral load to facilitate isolation in culture.

Rabbit kidney (RK-13) cells were used to isolate EHV-1, while African green monkey (Vero), Madin-Darby bovine kidneys (MDBK), equine dermal (ED) and primary equine lung (EEL) cells were used to isolate EHV-4. Cells were maintained in a 10 mL maintenance medium (supplemented with 8% FBS) at 37 °C in a 5% CO_2_ atmosphere. Twenty-five cm^3^ tissue culture flasks of near-confluent cells were inoculated with 0.5 mL tissue homogenate/nasal fluid. The cells were checked every day for signs of any cytopathic effect (CPE). Samples were considered negative if no CPE was observed after three passages for up to 7 days.

#### 2.2.2. Phylogenetic and Molecular Characterization

Two viruses—EHV-1/MA/2017/21 isolated from a nasal swab collected from a female donkey and EHV-1/MA/2010/21 isolated from the tissues of the aborted horse fetus described above—were submitted to the WOAH reference laboratory for genetic characterization.

Reduced multi-locus sequence typing (MLST) based on the loci of six open reading frames (ORFs), 11, 13, 30, 37, 52 and 76, of EHV-1 was performed [38] The PCR mixture (50 µL) for amplification of the target sequences consisted of 2.5 U GoTaq Hot Start Polymerase (5 U/µL) (Promega, Fitchburg, Wisconsin, USA) 1X GoTaq Flexi Buffer, 2 mM MgCl2 solution, 0.2 mM dNTP Mixture Mixture (Applied Biosystems, Vilnius, Lithuania), 0.4 of µM each primer, 5 µL of DNA, and nuclease-free water. The cycling conditions were as follows: initial denaturation at 95 °C for 5 min followed by 40 cycles of denaturation at 95 °C for 30 s, annealing at 57 °C for 45 s, elongation at 72 °C for 2 min and final extension at 72 °C for 5 min. PCR reactions were visualized on a 1.2% agarose gel stained with 0.0003% Sybersafe (Invitrogen by Thermofisher Scientific, California, USA) PCR amplicons were purified using the QIAquick PCR purification kit (Qiagen, Hilden, Germany, as per the manufacturer’s instructions). Sanger sequencing was performed by Eurofins Genomics, Cologne, Germany.

The nucleotide sequences obtained were aligned to relevant ORFs of EHV-1 reference strains Ab4 (GenBank accession AY665713.1) and V592 (GenBank accession AY464052.1) using the ClustalW [39] accessory application implemented in BioEdit sequence editor version 7.2.5 [40]. Nucleotide sequences of individual ORFs were translated in Bioedit and multiple amino acid sequence alignments were produced for each segment with Ab4 and V592 as reference sequences. Amino acid sequence differences were recorded and tabulated to complete MLST analysis.

Both viruses, EHV-1/MA/2010/21 and EHV-8/MA/2017/21 were submitted to PathoSense BV, Lier, Belgium for whole-genome sequencing as described previously [41]. The nucleotide sequences of two characterized strains in this study were submitted in the GenBank database under accession numbers PP839875.1 and PP839876.1, respectively, for the EHV-1 and EHV-8.

The genome sequences of the two Moroccan viruses were aligned with other equid alphaherpesvirus (EHV-1, EHV-3, EHV-4, EHV-8, and EHV-9) reference sequences using Bioedit and ClustalW (version 1.83). The accession numbers for the 50 viruses included are detailed in Supplementary Table S1. For the phylogenetic analysis, the relationship between the viruses was inferred using the Maximum Likelihood method conducted in the IQ-Tree software (version 1.6.12) [42]. The best-fit substitution model for each dataset was selected using the ModelFinder feature integrated into IQ-Tree, which evaluates a comprehensive set of models and selects the one with the lowest Bayesian Information Criterion (BIC) score. Data were bootstrapped 1000 times to assess the reliability of the phylogenetic tree produced. The resulting phylogenetic tree was visualized and annotated using iTOL [43]. The tree’s topology was analyzed to assess evolutionary relationships among the taxa.

A nucleotide sequence alignment of the UL of EHV-1/MA/2010/21 with the UL sequences of 40 EHV-1 genomes representative of the 13 clades identified by Bryant et al. [44] was performed using Mauve alignment tool [45]. The GenBank accession numbers of the included EHV-1 strains are detailed in Table S2. The PhyML extension for Geneious was used to create a maximum likelihood tree with 100 bootstrap replicates [46].

To assess the completeness and quality of genome assemblies and to identify conserved orthologous genes across the taxa, we employed the BUSCO [47] (Benchmarking Universal Single-Copy Orthologs) _phylogenomics pipeline (https://github.com/jamiemcg/BUSCO_phylogenomics, accessed on 7 May 2023), based on BUSCO genes in the class Herspesviridae. The BUSCO gene prediction was carried out by comparing 50 herpesvirus strains with the AHV-3 strain AR/2007/C3A as the outgroup.

The fastANI tool [48] (https://github.com/ParBLiSS/FastANI, accessed on 7 May 2023) was used to identify the percentage nucleotide identity of the complete Moroccan genome and the different genomes known in other countries, and the heat map was produced using R. The intra- and inter-group evolutionary distances of herpesviruses (EHV-1, EHV-8 and EHV4) were calculated using the maximum composite likelihood method. All positions containing gaps and missing data were removed.

## 3. Results

### 3.1. Molecular Prevalence

Real-time multiplex PCR was conducted to identify the presence of EHV-1 and EHV-4 in nasopharyngeal (NP) swabs and tissue samples, and to confirm suspected EHV-1 isolates.

Among the 154 samples tested, only 3 (1.94%) initially tested positive for EHV-1. These included an isolate from an aborted horse fetus. In fact, the fetal liver was inoculated in the RK13 cells, and the cytopathic effect on cell culture was suggestive of EHV-1 infection, although PCR confirmation was still necessary. Two additional NP swabs were collected—one from a donkey displaying respiratory symptoms and another from a mule exhibiting neurological symptoms. All three positive samples showed strong positivity, with Ct values of 20, 24 and 16, respectively.

For EHV-4, 42 samples (27%) tested positive, with horses exhibiting the highest prevalence (28.9%) compared with mules (22.58%) and donkeys (22.2%). The prevalence of EHV-4 varied among the geographic areas where the samples were collected. The regions of Fez-Meknes and Rabat recorded the highest prevalences (32%, 18/56 and 32.4%, 12/37, respectively), followed by Marrakech (24%, 8/33). The El Jadida region showed the lowest prevalence (14%, 4/28).

Among the EHV-4-positive equids, 64% (*n* = 10) were less than 2 years old, 33% (*n* = 11) were between 3 and 7 years old, and 24% (*n* = 5) were over 8 years old. The findings were also analyzed by gender, revealing that males had the highest prevalence, with 39.3% (*n* = 26) of positive samples, while only 18.2% (*n* = 17) of females tested positive (see Table 1).

The statistical analyses revealed that equid gender was the only statistically significant factor (*p* = 0.003), while the prevalence of species (*p* = 0.733), geographic region (*p* = 0.198) and age (*p* = 0.271) were not statistically significant (see Table 1).

Regarding the Ct values, three samples were highly positive, with Ct values of 17, 29, and 27. These samples corresponded to two NP samples from horses presenting respiratory symptoms and an abortion from a primiparous mare, respectively. Additionally, 19 samples had Ct values ranging between 30 and 35, all of which were associated with cases exhibiting respiratory symptoms. The remaining 20 samples had Ct values greater than 35, with 17 samples linked to respiratory signs, 2 samples obtained from aborted mares, and 1 from neonatal mortality.

### 3.2. Isolation and Sequencing

#### 3.2.1. Virus Isolation

Based on Ct < 30, virus isolation was performed on five samples. Two EHV-1 PCR-positive samples were cultured in RK13: one from a donkey with respiratory symptoms Ct = 24 and one from mule with neurological symptoms Ct = 16. For EHV-4, three samples had Ct < 30. They were inoculated on three different continuous cell lines. The three samples were from horses, two of which showed respiratory signs with a Ct of 17 and 29, while the final sample came from an aborted horse fetus with a Ct of 27. Attempts to isolate the virus from the three positive samples for EHV-4 were unsuccessful. Subsequent attempts to isolate the virus from EHV-4-positive samples on a primary equine embryonic cell line at the WOAH reference laboratory were also unsuccessful.

**Figure 1:**
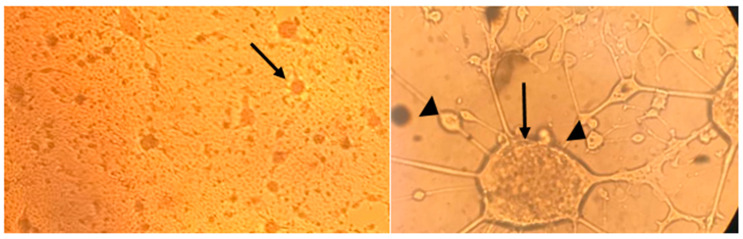
Cytopathogenic effect of EHV-1 (the respiratory donkey) on RK-13 cells, showing syncytium formation (arrows) and ballooning degeneration (arrowheads). The left image (40 × magnification) provides an overview of the cytopathic effects, while the right image (200 × magnification) offers a detailed view of the cellular alterations.

However, the EHV-1 PCR-positive sample from the donkey tested positive in cell culture, showing a CPE characterized by rounded cells and syncytia (Figure 1). This sample was further characterized through sequencing and phylogenetic analysis, along with the 2010 aborted horse fetus isolate from the Biopharma laboratory in Morocco, which was also confirmed by PCR as part of our study. Sequencing and phylogenetic analysis were performed at the WOAH Reference Laboratory.

#### 3.2.2. Phylogenetic Analysis and Molecular Characterization

**Figure 2:**
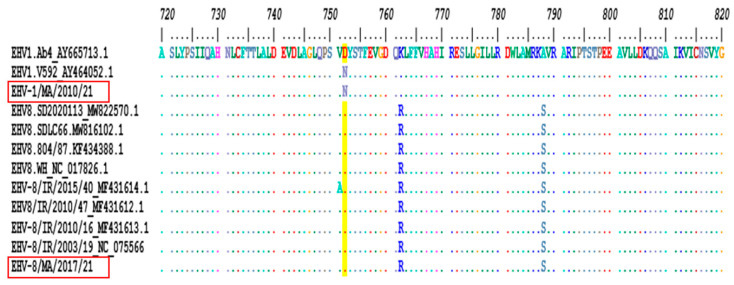
Alignment of the predicted amino acid sequence of ORF30 polymerase gene for EHV-1 and EHV-8 viruses including the two Moroccan viruses. Amino acid positions are indicated above the alignment. Identical residues are indicated with a dot. Amino acid residue D752 corresponds to the proposed marker of neuropathogenicity in EHV-1 (highlighted in yellow). The Moroccan strains are framed in red.

The EHV-1 PCR assay targeting the glycoprotein C gene performed at the WOAH reference laboratory detected EHV-1 in the EHV1/MA/2010/21 sample at a Ct value of 14, similar to the Diallo assay. However, for the donkey sample initially designated EHV-1/MA/2017/21, the Ct value of 37 was significantly higher than that detected with the Diallo assay (Ct = 22). Subsequently, MLST analysis showed that the sequence derived from a 461-nucleotide segment of the ORF30 of this virus shared more of its nucleotide identity with EHV-8 strains (99.57% to 100%) compared to EHV-1 (sharing 94.36% identity with V592 and 94.57% with Ab4). The closer resemblance to EHV-8 was confirmed in all other segments sequenced for MLST In contrast, the MLST of EHV-1/MA/2010/21 using 13 amino acid differences in six open reading frames (ORFs) identified it as a clade 1 EHV-1 virus. These amino acid differences are summarized in Table 2. Analysis of the viral polymerase gene ORF30 identified the equine virus EHV-1/MA/2010/21 as an A2254/N752 genotype and the donkey virus as a G2254/D752 genotype (Figure 2).

**Figure 3:**
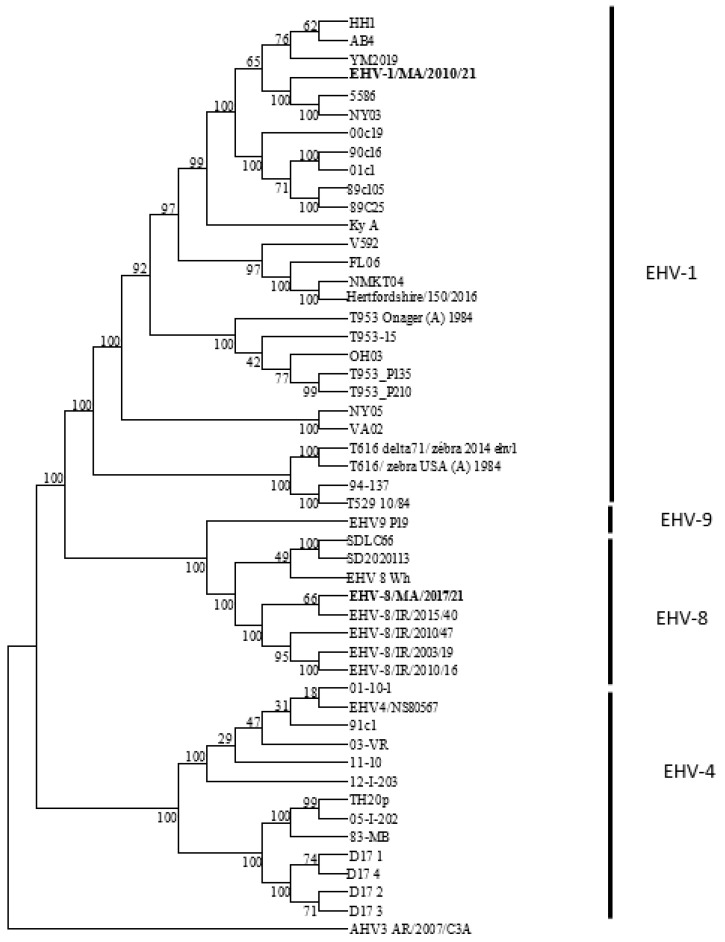
Maximum-likelihood phylogenetic tree (scale bar approximately 0.02 substitutions per site), indicating greater evolutionary distance or divergence among equid alphaherpesvirus strains, including the two Moroccan viruses sequenced in this study (highlighted in bold). The analysis included 50 genome sequences, and bootstrap values calculated from 1000 replicates are shown at major nodes.

The analysis of the whole genome sequence of the two samples unequivocally identified the isolate from the donkey as an EHV-8 strain (renamed EHV8/MA/2017/21) and the isolate from the aborted mare fetus as an EHV-1 strain (EHV1/MA/2010/21). Phylogenetic analysis (see Figure 3) revealed that the genome of EHV-1/MA/2010/21 is closely related to the genomes of the EHV-1 strains HH1, Ab4, YM2019, 5586, and NY03. In contrast, EHV-8/MA/2017/21 is more closely related to EHV-8 strains, including EHV-8/IR/2003/19 (equine), EHV-8/IR/2010/16 (asinine), EHV-8/IR/2010/47 (equine) and, in particular, EHV-8/IR/2015/40 (asinine). Additionally, EHV-8/MA/2017/21 is more closely related to EHV-9 than to EHV-1 based on the phylogenetic analysis (see Figure 3).

**Figure 4:**
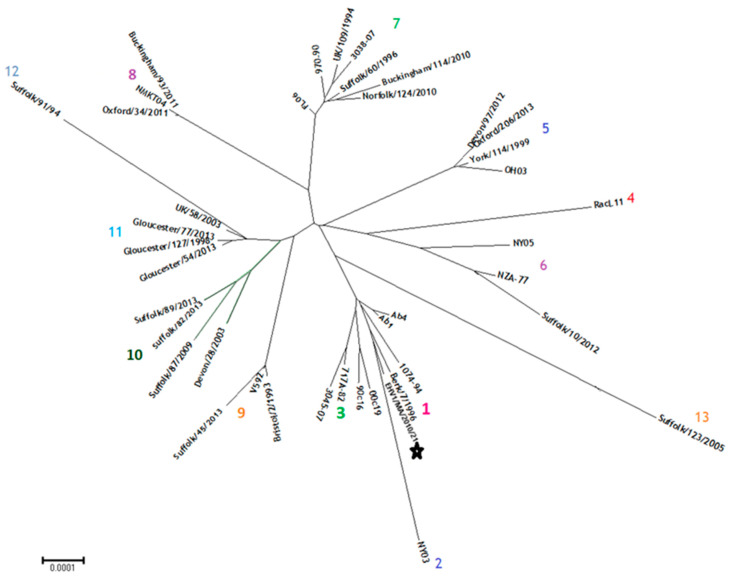
Phylogenetic analysis of EHV-1 genomes from 13 distinct UL clades (numbered 1 to 13, with each clade represented by a different color), including the Moroccan strain EHV1/MA/2010/21 sequenced in this study, highlighted with a star. The scale bar represents the number of nucleotide substitutions per site.

Further phylogenetic analysis of the UL region of strain EHV-1/MA/2010/21 with 40 representative sequences of the 13 UL clades showed that EHV-1/MA/2010/21 clustered with UK strain Berkshire/7/1996, 1074-94, Ab4 and Ab1 (Figure 4).

The sequence homology analysis for the two Moroccan viruses revealed 91.17% shared identity in the predicted protein sequences. The EHV-1/MA/2010/21 strain was more closely related to other EHV1 strains, including HH1, Ab4, NY03 and 90c16 (99.84–99.82%) (Table S1). The EHV-8/MA/2017/21 protein homology to various Irish EHV-8 strains, including EHV-8/IR/2003/19, EHV-8/IR/2010/16, EHV-8/IR/2010/47 and EHV-8/IR/2015/40, was 99.75–99.70%. However, the protein homology was lower when compared to the reference EHV-8Wh strain (99.45%) (Table S2). The Moroccan viruses shared the lowest identity (93.60–93.72%) with the EHV-9 P19 virus strain.

The genetic diversity between virus strains is summarized in Table S4. The diversity between the EHV-1 and EHV-8 viruses isolated in Morocco was estimated to 0.16. However, the divergence between the EHV-1 virus isolated in Morocco and other international EHV-1 strains varied between 0.05 and 0.172. EHV-1/MA/2010/21 was closest to the Hertfordshire/150/2016 strain detected in England, while it exhibited the longest distance from the KyA strain. The analysis of the distance between EHV-8/MA/2017/21 and the other EHV-8 viruses sequenced worldwide showed that the EHV-8/MA/2017/21 was the closest to the SD2020113 strain and the furthest from the YM2019 strain, with genetic distances of 0.093 and 0.25, respectively. The distance from the four Irish strains was 0.147, while the distance from the EHV-8 Wh strain was 0.24. EHV-9 P19 had smaller genetic distance from EHV-1/MA/2010/21, with a value of 0.189, compared to EHV-8/MA/2017/21, which was 0.21 (Table S4).

## 4. Discussion

This study marks the first demonstration of the presence of EHV-1, EHV-4 and EHV-8 in Moroccan equidae utilizing PCR. It is also the first to report the detection of EHV-8 in a donkey in Africa and contains the first molecular characterization of this virus and a Moroccan EHV1 isolate.

### 4.1. Molecular Prevalence

A multiplex real-time PCR, targeting the highly conserved glycoprotein B region, was used for the identification and differentiation of EHV-1 and EHV-4 herpesviruses [24,36]. Samples were collected from clinical cases exhibiting respiratory and neurological symptoms, as well as cases of abortions and neonatal deaths.

EHV-1 was detected in 3 out of 154 samples (1.94%), including a donkey and a mule displaying respiratory and neurological signs, respectively. Additionally, the study confirmed the presence of EHV-1 in an aborted horse fetus from a mare isolated in 2010.

Molecular investigations into clinically ill equines have been conducted in various countries, revealing diverse prevalence rates. Comparable prevalence rates to our study were reported in Algeria (2%) [33] and in the United States (2.7%) [49]. Conversely, higher prevalence rates were documented in other studies, such as 3.69% in Tunisia [34], 5.6% in South Korea [50] and 7.5% in Ethiopia [51].

Epidemiological studies examining the molecular prevalence of EHV-1 in healthy horses have also been undertaken. These studies indicate notably higher prevalence levels compared to our findings. For instance, a study involving 1497 healthy horses in South Korea reported a prevalence of 12.0% [52]. Additionally, an investigation conducted on 90 asymptomatic horses in two regions of Iran revealed EHV-1 prevalence ranging from 8.10% to 18.18% [53].

The overall prevalence of EHV-4 in our study was 27.3% (42/154). This frequency was notably higher than the rates reported in healthy equines from other countries. In Iran, the prevalence ranges from 16.21% to 16.98% [53]. Moreover, our findings indicate an even greater prevalence when compared to studies conducted among animals exhibiting clinical symptoms, such as in South Korea (7.9%) [50] in Ethiopia (8.1%) [51], in the United States (8.4%) [49], and in Algeria (12%) [33]. These epidemiological data strongly suggest the widespread distribution of the virus in Morocco.

EHV-4 was detected in the aborted fetuses of three horses and in one case of neonatal mortality. Unlike EHV-1, EHV-4 is rarely associated with abortion in horses and is primarily confined to the respiratory system. Izume et al. [54] aimed to identify the key genes of the EHV-4 genome responsible for abortion in female horses. They analyzed the full genome sequences and predicted amino acid sequences of eight EHV-4 isolates, including two from aborted fetuses and five from nasal swabs of horses with respiratory disease. However, their study did not identify any genes associated with EHV-4-induced abortion. The actual incidence of abortion caused by this virus in the field is not well established [54]. Reported rates were less than 1% in Kentucky, USA, between 1983 and 1992, and up to 16% in England between 1987 and 1993 [55]. EHV-4 was also detected in a nasal swab from an aborted fetus in Turkey [56].

The observed disparity between the two *Alphaherpesviruses* in terms of the frequency between the two viruses underscores the higher prevalence and transmission rates of EHV-4 compared to EHV-1. Previously, EHV-4 was found to be more prevalent during outbreaks of respiratory disease [17]. Notably, in 2017, EHV-4 played a pivotal role in a significant epizootic outbreak in northern Germany, affecting foals, mares and stallions [57]. Unlike EHV-1, EHV-4 can be expressed in equines throughout the year, with no discernible link to seasonal variation [58], indicating that the virus is frequently reactivated and excreted throughout the life of the equine.

Also, the elevated molecular prevalence of EHV-4 aligns with findings from a seroprevalence investigation involving 405 horses in Morocco in 2021 [59]. This serological study, employing a type-specific ELISA, reported a prevalence of 12% for EHV-1 and 100% for EHV-4. Remarkably, this prevalence held true across different risk factors, including age, gender, area, breed and activity. Seroprevalence studies in donkeys and mules have similarly demonstrated a higher seroprevalence of EHV-4 compared to EHV-1 [60,61].

A statistical analysis was conducted to assess the intrinsic factors, such as age, gender, and species, associated with the prevalence of EHV-4. Interestingly, no statistically significant difference was found concerning the age of the equid, indicating that all age groups were susceptible to EHV-4 infection. However, the highest prevalence was observed in equids aged less than 2 years. This aligns with similar findings in the studies of Negussie et al. [51] and Laabassi et al. [33], in which the greatest prevalence was identified in young horses, particularly yearling foals. According to Gilkerson et al. [17], this could be related to the severity of clinical symptoms in young horses. Additionally, Foot et al. [62] demonstrated that early infections are likely a result of foals’ direct contact with their mothers during the first months of life, followed by horizontal transmission between foals. In contrast, in their research, Taktaz Hafshejani et al. [53] found that adult/senior horses were the most susceptible.

Gender has a considerable effect on virus prevalence, according to our statistical analyses. Males were more likely to be infected than females. The same results were reported in an Ethiopian investigation by Negussie et al. [51], as well as an Iranian study by Taktaz Hafshejani et al. [53] in the Isfahan region. Taktaz Hafshejani et al. [53] explained this difference through the argument that males are used significantly more than females in reproduction (a single stallion may be mated with multiple mares) and equestrian sports. However, in our research, the majority of our samples were from draft equids, with no significant differential of usage based on sex.

Similarly, horses demonstrated a higher prevalence compared to mules and donkeys, although the difference was statistically insignificant. In contrast, Negussie et al. [51] showed that in Ethiopia, donkeys exhibited the highest frequency of prevalence. In addition, according to Lara et al. [63], donkeys can be an active carrier of these diseases and they can spread infections to their cohort. Finally, Pusterla et al. [64] emphasize the role of mules as silent shedders. This highlights the importance of implementing appropriate biosecurity measures whenever horses and other household equids come into contact.

The analysis of viral concentration based on the Ct value revealed that a significant proportion of positive samples (79.5%) exhibited only weak positivity (Ct > 35). The Ct value serves as an indicator of the viral load in respiratory tract excretion and can be affected by factors such as immune response, age, infectious stage, strain, and viral load [50,65]. An elevated Ct value could indicate either latent infection or recovery from an acute infection [66] and it was demonstrated that the amount of virus detected in nasal samples can change over time [67]. Furthermore, Pusterla et al. [64] showed that the glycoprotein B gene used in our PCR can detect EHV-4 up to 4 weeks after infection.

Despite the fact that all of the samples were obtained from animals showing clinical signs, 69% of samples were negative. This finding can be explained by the moment of sampling with regard to the viral excretion, as well as the possibility of false negatives due to the difficulty of obtaining nasal pharyngeal swabs [68]. However, the clinical symptoms observed can be explained by the presence of other infections, such as equine influenza, West Nile virus, viral arteritis, or bacteria such as *Lepstospira interrogans*, *Streptococcus equi subsp zooepidemius*, *Escherichia coli*, *Klebsiella* sp., *Pseudomonas* sp. and *Salmonella* spi., all of which require confirmation via a specific PCR test.

### 4.2. Isolation in Cell Culture

The isolation of EHV-1 and EHV-4 from clinical samples was performed for samples with Ct < 30. To isolate EHV-1, we utilized RK13 (rabbit kidney-13) cells, a continuous cell line known for its suitability in such isolations [20]. These cells have been widely and successfully used in various studies [38,41,66,69,70,71] for the isolation of EHV-1. Successful virus isolation was achieved from the donkey sample exhibiting respiratory symptoms, whereas isolation from the mule sample presenting with neurological symptoms was unsuccessful.

However, attempts to isolate EHV-4 have been made using various cell types, including equine cell lines such as equine fibroblasts (EDs), as well as non-horse-derived continuous cell lines like Vero and MDBK cells. Interestingly, no expression of the EHV-4 virus was observed in our study across the different cell types used, which aligns with findings from other studies utilizing distinct cell types, including EEL, MDBK and RK13 cells [56,72]. On the contrary, successful isolation of EHV-4 was reported in studies utilizing equine cells such as ED cells [57,73] and non-equine lines like Vero cells [66,69] and MDBK cells [74].

The failure to isolate EHV-4 in our study can be attributed to several factors, including cell culture type, viral characteristics, sample quality and viral load in clinical samples. Unlike EHV-1, which can infect both equine and non-equine cells, EHV-4 has a preference for equine cells [20]. However, the sensitivity of cells or cell lines can vary significantly, potentially leading to false-negative results [20]. Unfortunately, the unavailability of specific cell lines or primary equine cells in Morocco, coupled with the impracticality of preparing primary cells from embryos, posed challenges with regard to conducting the necessary isolation experiments.

Another characteristic of herpesviruses, particularly EHV-1, that might account for the failure to isolate is the potential presence of interferon-alpha (INF-α) in nasal secretions, which is known to limit viral replication [71,75]. On the other hand, Pavulraj et al. (2021) observed during an EHV-4 outbreak that only samples with Ct values less than 25 could be isolated in cell culture. In our study, despite some samples testing strongly positive for EHV-4 (Ct = 17) and EHV-1 (Ct = 16), isolation in cell cultures proved unsuccessful. This discrepancy between PCR and viral isolation results can be explained by the fact that PCR can detect both infectious virus and non-infectious viral DNA, whereas viral isolation specifically requires live viruses [20].

False-negative results may also arise from postmortem changes. Some tissues contain enzymes that are toxic to cell cultures and may also contain viral inhibitors that interfere with the viral isolation procedure [76]. Therefore, a positive PCR result does not necessarily imply the presence of infectious virus in a clinical sample, which may elucidate the isolation failure despite the high viral load detected by PCR.

### 4.3. Phylogenetic Analysis and Molecular Characterization

The complete genome sequences of two Moroccan viruses were determined through Illumina sequencing and compared with Alphaherpesvirus genomes available in GenBank, including EHV-1, EHV-3, EHV-8, EHV-9, and EHV-4. Phylogenetic analysis revealed their close relationship to EHV-9, with less similarity to EHV-4.

The EHV8/MA/2017/21 strain isolated from a donkey was initially misdiagnosed as EHV-1 due to the close genetic relationship between these viruses. Misdiagnosis of EHV-8 was described previously by Garvey et al. [77] when two EHV-8 strains were isolated from cases of abortion in horses and were initially incorrectly identified as EHV-1 due to cross-reactivity of the PCR assay [77]. Reanalysis of the Moroccan samples with a more specific PCR assay raised concern, and further sequence analysis identified the donkey virus as EHV-8 (EHV-8/MA/2017/21). Comparative analysis of the genome sequence of EHV-8/MA/2017/21 with other EHV-8 strains revealed its close relationship to Irish strains isolated from both donkeys and horses [77]. Initially identified in 1988 in Australia, as asinine herpesvirus 3 (AHV3), EHV-8 was first isolated from donkeys treated with high doses of corticosteroids, resulting in rhinitis [78]. The infected donkey in this study presented with respiratory signs, but recent evidence from the Netherlands, Ireland and China suggests that EHV-8 can also cause neurological disease [77,79,80] and abortion in donkeys [80]. As donkeys play an important economic role in Morocco, particularly in transport and agriculture, with a population of 891,000 [81], EHV-8-associated diseases could significantly impact livelihoods in these communities. The potential role of donkeys as reservoirs of EHV-8 and the virus’s ability to cross species barriers [77] underscore the need for further investigation and its inclusion in the differential diagnosis of abortion and neurological diseases in equids. Furthermore, interspecies contact should be carefully regulated using evidence-based epidemiological practices to mitigate cross-species transmission and minimize the risk of outbreaks.

In contrast, EHV-1 is recognized as a significant pathogen in Morocco, where there is mandatory vaccination of breeding horses. A recent seroprevalence study found that 12.8% of unvaccinated and 21.8% of vaccinated horses tested positive for EHV-1 [59]. This study also reports the first genetic characterization of a Moroccan EHV-1 isolate (EHV-1/MA/2010/21) by MLST and whole-genome sequencing. Following the determination of the genome sequences of neuropathogenic strain Ab4 [30] and non-neuropathogenic strain V592 [82], interest in EHV-1 strain differentiation increased. Bryant et al. [44] classified EHV-1 into 13 distinct clades using next-generation sequencing (NGS) analysis of 78 UK strains and 26 EHV-1 strains from other countries. Garvey et al. [38] developed a MLST approach for rapid clade assignment and molecular epidemiological investigations. The approach was subsequently used to explore the genetic diversity of viruses in France and Belgium [71,83] and to characterize the virus linked to the outbreak of neurological disease in the Iberian peninsula in 2021 [84]. MLST analysis in this study placed EHV-1/MA/2010/21 in clade 1, which is associated with neurological disorders and hypervirulence [38]. A study by Nugent et al. [82] identified a single amino acid variation in the DNA polymerase gene (D752 vs. N752) as being strongly associated with neurological vs. non-neurological disease outbreaks. EHV-1/MA/2010/21 was identified as an N752 genotype, while the putative neurological marker (D752) was found in EHV-8/MA/2017/21, consistent with other sequenced EHV-8 strains. Although D752 strains show higher replication rates and prolonged viremia [85,86], neurological disease cannot be fully attributed to this polymorphism, as not all neurological cases are linked to D752 strains [87,88,89,90]. Phylogenetic analysis confirmed that EHV-1/MA/2010/21 is closely related to abortigenic strains from the UK (Berkshire/7/1996), Japan (HH1), the USA (NY03) and China (YM) [44], showing limited genetic diversity in global EHV-1 strains. Similarly, EHV-8/MA/2017/21 displayed a close genetic relationship to EHV-8 strains from Asia and Europe.

## 5. Conclusions

This study highlighted the active circulation of EHV-1 and EHV-4 in the Moroccan equine population and the first detection of EHV-8 in a donkey in Africa. It also showed that EHV-4 is more prevalent and can be involved not only in respiratory disorders but also in abortions and neonatal deaths. Molecular characterization of EHV-1 and EHV-8 Moroccan isolates was performed for the first time. MLST and complete genome analysis of the Moroccan EHV-1 strain showed that it belongs to UL clade 1, where strains are characterized by their hypervirulence. Meanwhile, the Moroccan isolate of EHV-8 was genetically close to Irish strains isolated from donkeys with respiratory disease and abortion in horses. Therefore, EHV-8/MA/2017/21 could represent a potential risk to horses in Morocco. These findings reinforce that health precautions must be taken during equid interspecies gatherings.

## Figures and Tables

**Table 1:** Statistical analysis of the effect of different variables on EHV-4 positivity.

	Category	Negative (%)	Positive (%)	X^2^–Value	*p*–Value
**Areas**	Casablanca	85.71	14.29	4.664	0.198
	Fes–Meknes	71.43	28.57		
	Marrakech	75.76	24.24		
	Rabat	62.16	37.84		
**species**	Donkey	77.78	22.22	0.621	0.733
	Horse	71.05	28.95		
	Mule	77.42	22.58		
**Sex**	Female	81.81	18.18	8.556	0.003 *
	Male	60.61	39.30		
**Age (years)**	1–2	60.87	39.13	2.609	0.271
	3–7	75.00	25.00		
	≥8	80.77	19.23		

* Significance of *p* value.

**Table 2:** Multi-locus sequence analysis of six open reading frames (ORFs). Shading is used to highlight amino acid difference at that position. The proposed marker of neuropathogenicity N752/D752 in EHV-1 ORF 30 is highlighted in yellow.

		ORF And Amino Acid Position												
**Virus**	**UL Clade**	**11** ^1^	**11**	**13**	**13**	**13**	**13**	**13**	**13**	**30**	**30**	**37**	**52**	**76**
		189 ^2^	235	305	405	460	492	493	499	752	990	265	386	128
EHV1 AB4	1	Q	R	S	A	A	E	T	A	D	E	A	A	F
EHV1/MA/2010/21	1	Q	R	S	A	A	E	T	A	N	E	A	A	F
V592	9	K	R	L	A	T	E	T	A	N	K	V	V	S

^1^ ORF number. ^2^ Amino acid position.

## Data Availability

The original contributions presented in this study are included in the article/supplementary material. Further inquiries can be directed to the corresponding author.

## Supplementary Materials

The following supporting information can be downloaded at https://www.mdpi.com/article/10.3390/ani15010102/s1. Table S1: Percentage of nucleotide similarity between the EHV-1/MA/2010/21 strain and the reference strains. Table S2: EHV-1 Reference sequences from 13 UL clades. Table S3: Percentage of similarity between the EHV-8/MA/2017/21 strain and the reference strains. Table S4: Genetic diversity between EHV-1/MA/2010/21, EHV-8/MA/2017/21 and different reference strains.
