# Supplementary material for: First Molecular and Phylogenetic Characterization of Equine Herpesvirus-1 (EHV-1) and Equine Herpesvirus-4 (EHV-4) in Morocco
Source: Animals (Basel). 2025 Jan 5;15(1):102. doi: 10.3390/ani15010102 (PMC11718982; doi:10.3390/ani15010102)
Supplement: Supplementary file 1 [file animals-15-00102-s001.zip › Table S1.pdf]

Table S1: Percentage of nucleotide similarity between the EHV-1/MA/2010/21 strain and the reference strains

	Strains	GenBank accession number	%
EHV-1/MA/2010/21	EHV-1/MA/2010/21	PP839875.1	100
EHV-1/MA/2010/21	EHV1/HH1	AB992258.1	99.844
EHV-1/MA/2010/21	EHV1/Ab4	AY665713.1	99.8375
EHV-1/MA/2010/21	EHV1/NY03	KF644569.1	99.8284
EHV-1/MA/2010/21	EHV1/90c16	KF644566.1	99.8227
EHV-1/MA/2010/21	EHV1/Hertfordshire/150/2016	KY852346.1	99.8216
EHV-1/MA/2010/21	EHV1/ YM2019	MT063054.1	99.8164
EHV-1/MA/2010/21	EHV1/89c105	KF644577.1	99.8124
EHV-1/MA/2010/21	EHV1/01c1	KF644578.1	99.8057
EHV-1/MA/2010/21	EHV1/00c19	KF644576.1	99.8052
EHV-1/MA/2010/21	EHV1/89c25	KF644579.1	99.8038
EHV-1/MA/2010/21	EHV1/NY05	KF644570.1	99.7873
EHV-1/MA/2010/21	EHV1/VA02	KF644572.1	99.7862
EHV-1/MA/2010/21	EHV1/ V592	AY464052.1	99.7817
EHV-1/MA/2010/21	EHV1/NMKT04	KF644568.1	99.7673
EHV-1/MA/2010/21	EHV1/FL06	KF644567.1	99.7657
EHV-1/MA/2010/21	T953_P15	KP975078.1	99.7651
EHV-1/MA/2010/21	EHV1/T953	KM593996.1	99.7648
EHV-1/MA/2010/21	EHV1/ T953_P135	KR021354.1	99.7621
EHV-1/MA/2010/21	EHV1/T953_P210	KR047045.1	99.7557
EHV-1/MA/2010/21	EHV1/OH03	KF644571.1	99.7425
EHV-1/MA/2010/21	EHV1/KyA	MF975655.1	99.6551
EHV-1/MA/2010/21	EHV1/5586	AP012321.1	99.6107
EHV-1/MA/2010/21	EHV1/94-137	KF644575.1	97.2425
EHV-1/MA/2010/21	EHV1/T-529 10/84	KF644580.1	97.2424
EHV-1/MA/2010/21	EHV1/ T-616	KF644574.1	97.0937
EHV-1/MA/2010/21	EHV1/T616 delta71	KF644573.1	97.0796
EHV-1/MA/2010/21	EHV-9 P19	AP010838.1	93.6028
EHV-1/MA/2010/21	EHV-8 Wh	JQ343919.1	91.6102
EHV-1/MA/2010/21	EHV-8/IR/2010/16	MF431613.1	91.465
EHV-1/MA/2010/21	EHV-8/IR/2015/40	MF431614.1	91.465
EHV-1/MA/2010/21	EHV-8/IR/2010/47	MF431612.1	91.465
EHV-1/MA/2010/21	EHV-8/IR/2003/19	MF431611.1	91.3851
EHV-1/MA/2010/21	EHV-8/SDLC66	MW816102.1	91.3429
EHV-1/MA/2010/21	EHV-8/SD2020113	MW822570.1	91.3181
EHV-1/MA/2010/21	EHV-8/MA/2017/21	PP839906.1	91.1764
EHV-1/MA/2010/21	EHV-4/03-VR	LC075585.1	80.8572
EHV-1/MA/2010/21	EHV-4/91c1	LC075583.1	80.755
EHV-1/MA/2010/21	EHV-4/11 10	LC075587.1	80.747
EHV-1/MA/2010/21	EHV-4/DE17_2	MW892436.1	80.7449
EHV-1/MA/2010/21	EHV4/NS80567	NC_001844.1	80.741
EHV-1/MA/2010/21	EHV-4/DE17_4	MW892438.1	80.7381

EHV-1/MA/2010/21	EHV-4/DE17_1	MW892435.1	80.7297
EHV-1/MA/2010/21	EHV-4/DE17_3	MW892437.1	80.7153
EHV-1/MA/2010/21	EHV-4/12-1-203	LC075588.1	80.7011
EHV-1/MA/2010/21	EHV-4/05-1-202	LC075586.1	80.6131
EHV-1/MA/2010/21	EHV-4/83-MB	LC075582.1	80.6082
EHV-1/MA/2010/21	EHV-4/01 10 2001	LC075584.1	80.5814
EHV-1/MA/2010/21	EHV-4/TH20p	LC063142.1	80.5739
EHV-1/MA/2010/21	AHV-3 AR/2007/C3A	KM051845.1	75.7614
