# Supplementary material for: First Molecular and Phylogenetic Characterization of Equine Herpesvirus-1 (EHV-1) and Equine Herpesvirus-4 (EHV-4) in Morocco
Source: Animals (Basel). 2025 Jan 5;15(1):102. doi: 10.3390/ani15010102 (PMC11718982; doi:10.3390/ani15010102)
Supplement: Supplementary file 1 [file animals-15-00102-s001.zip › Table S2.pdf]

Table S2 : EHV-1 Reference Sequences from 13 UL clades

EHV-1 Strain ID	GenBank Accession Number
00c19	KF644576.1
1074-94	KT324730.1
3038-07	KT324726.1
3045-07	KT324725.1
717A-82	KT324733.1
90c16	KF644566.1
970-90	KT324732.1
Ab1	KU206468.1
Ab4	AY665713.1
Berk/7/1996	KU206463.1
Bristol/2/1993	KU206451.1
Buckingham/114/2010	KU206433.1
Buckingham/93/2011	KU206455.1
Devon/28/2003	KU206440.1
Devon/97/2012	KU206469.1
FL06	KF644567.1
Gloucester/127/1998	KU206445.1
Gloucester/54/2013	KU206447.1
Gloucester/77/2013	KU206446.1
NMKT04	KF644568.1
Norfolk/124/2010	KU206427.1
NY03	KF644569.1
NY05	KF644570.1
NZA-77	KT324724.1
OH03	KF644571.1
Oxford/206/2013	KU206470.1
Oxford/34/2011	KU206456.1
RacL11	KU206478.1
Suffolk/10/2012	KU206474.1
Suffolk/123/2005	KU206480.1
Suffolk/45/2013	KU206452.1
Suffolk/60/1996	KU206422.1
Suffolk/82/2013	KU206441.1
Suffolk/87/2009	KU206443.1
Suffolk/89/2013	KU206442.1
Suffolk/91/94	KU206479.1
UK/109/1994	KU206434.1
UK/58/2003	KU206444.1
V592	AY464052.1
York/114/1999	KU206473.1
