# Supplementary material for: First Molecular and Phylogenetic Characterization of Equine Herpesvirus-1 (EHV-1) and Equine Herpesvirus-4 (EHV-4) in Morocco
Source: Animals (Basel). 2025 Jan 5;15(1):102. doi: 10.3390/ani15010102 (PMC11718982; doi:10.3390/ani15010102)
Supplement: Supplementary file 1 [file animals-15-00102-s001.zip › Table S3.pdf]

Table S3: Percentage of similarity between the **EHV-8/MA/2017/21** strain and the reference strains

	Strains	GenBank Accession number	%
EHV-8/MA/2017/21	EHV-8/MA/2017/21	PP839876.1	100
EHV-8/MA/2017/21	EHV-8/IR/2003/19	MF431611.1	99.7586
EHV-8/MA/2017/21	EHV-8/IR/2010/16	MF431613.1	99.7503
EHV-8/MA/2017/21	EHV-8/IR/2015/40	MF431614.1	99.7486
EHV-8/MA/2017/21	EHV-8/IR/2010/47	MF431612.1	99.7074
EHV-8/MA/2017/21	EHV-8 SDLC66	MW816102.1	99.659
EHV-8/MA/2017/21	EHV-8 SD2020113	MW822570.1	99.6533
EHV-8/MA/2017/21	EHV-8 Wh	JQ343919.1	99.4579
EHV-8/MA/2017/21	EHV-9 P19	AP010838.1	93.7206
EHV-8/MA/2017/21	EHV-1 T-616	KF644574.1	91.9342
EHV-8/MA/2017/21	EHV-1 T616 delta71	KF644573.1	91.8957
EHV-8/MA/2017/21	EHV-1 94-137	KF644575.1	91.8009
EHV-8/MA/2017/21	EHV-1 T-529 10/84	KF644580.1	91.7585
EHV-8/MA/2017/21	EHV-1 Hertfordshire/150/2016	KY852346.1	91.4736
EHV-8/MA/2017/21	EHV-1 NMKT04	KF644568.1	91.4172
EHV-8/MA/2017/21	EHV-1 OH03	KF644571.1	91.4013
EHV-8/MA/2017/21	EHV-1 Ab4	AY665713.1	91.3794
EHV-8/MA/2017/21	EHV-1 HH1	AB992258.1	91.368
EHV-8/MA/2017/21	EHV-1 01c1	KF644578.1	91.3417
EHV-8/MA/2017/21	EHV-1 FL06	KF644567.1	91.3396
EHV-8/MA/2017/21	EHV-1 90c16	KF644566.1	91.3333
EHV-8/MA/2017/21	EHV-1 00c19	KF644576.1	91.3298
EHV-8/MA/2017/21	EHV-1 89c105	KF644577.1	91.329
EHV-8/MA/2017/21	EHV-1 NY03	KF644569.1	91.3288
EHV-8/MA/2017/21	EHV-1 VA02	KF644572.1	91.3139
EHV-8/MA/2017/21	EHV-1 NY05	KF644570.1	91.283
EHV-8/MA/2017/21	EHV-1/MA/2010/21	PP839875.1	91.2799
EHV-8/MA/2017/21	EHV-1 89c25	KF644579.1	91.2664
EHV-8/MA/2017/21	EHV-1 T953	KM593996.1	91.264
EHV-8/MA/2017/21	EHV-1 YM2019	MT063054.1	91.2537
EHV-8/MA/2017/21	EHV-1/ KyA	MF975655.1	91.2523
EHV-8/MA/2017/21	EHV-1/T953_P210	KR047045.1	91.2486
EHV-8/MA/2017/21	EHV1 génome complet	KP975078.1	91.2457
EHV-8/MA/2017/21	EHV-1 /V592	AY464052.1	91.2421
EHV-8/MA/2017/21	EHV-1 /T953_P135	KR021354.1	91.2366
EHV-8/MA/2017/21	EHV-1/5586	AP012321.1	91.1522
EHV-8/MA/2017/21	EHV-4 /DE17_2	MW892436.1	80.6808
EHV-8/MA/2017/21	EHV-4 /DE17_4	MW892438.1	80.6808
EHV-8/MA/2017/21	EHV-4/ 03-VR	LC075585.1	80.6673
EHV-8/MA/2017/21	EHV-4 /12-1-203	LC075588.1	80.662
EHV-8/MA/2017/21	EHV-4 /TH20p	LC063142.1	80.6614
EHV-8/MA/2017/21	EHV-4 /83-MB	LC075582.1	80.6528

EHV-8/MA/2017/21	EHV-4 /DE17_3	MW892437.1	80.6353
EHV-8/MA/2017/21	EHV-4 /DE17_1	MW892435.1	80.6353
EHV-8/MA/2017/21	EHV-4 /05-1-202	LC075586.1	80.6256
EHV-8/MA/2017/21	EHV-4 /01 10 2001	LC075584.1	80.611
EHV-8/MA/2017/21	EHV-4 /91c1	LC075583.1	80.5871
EHV-8/MA/2017/21	EHV-4 /11 10	LC075587.1	80.5689
EHV-8/MA/2017/21	EHV4/NS80567	NC_001844.1	80.5581
EHV-8/MA/2017/21	AHV-3 AR/2007/C3A	KM051845.1	76.111
