# Supplementary material for: First Molecular and Phylogenetic Characterization of Equine Herpesvirus-1 (EHV-1) and Equine Herpesvirus-4 (EHV-4) in Morocco
Source: Animals (Basel). 2025 Jan 5;15(1):102. doi: 10.3390/ani15010102 (PMC11718982; doi:10.3390/ani15010102)
Supplement: Supplementary file 1 [file animals-15-00102-s001.zip › Table S4.pdf]

Table S4: Genetic diversity between EHV-1/MA/2010/21, EHV-8/MA/2017/21 and different reference strains.

				1	2	3	4	5	6	7	8	9	10	11	12	13	14	15	16	17	18
			N° Gene Bank	AP010838.1	AP012321.1	AY464052.1	AY665713.1	**PP839875.1**	**PP839876.1**	JQ343919.1	KY852346.1	LC063142.1	MF431611.1	MF431612.1	MF431613.1	MF431614.1	MF975655.1	MT063054.1	MW816102.1	MW822570.1	NC_001844.1
1	EHV-9	P19	AP010838.1	0	0.226845	0.223173	0.225277	0.189896	0.21087	0.234657	0.145958	0.514498	0.230274	0.231486	0.229781	0.231148	0.256233	0.225271	0.22769	0.227666	0.515115
2	EHV-1	5586	AP012321.1	0.226845	0	0.167964	0.170318	0.133556	0.258709	0.27544	0.0913324	0.524651	0.275897	0.277591	0.275433	0.276679	0.202955	0.170127	0.273769	0.273739	0.525439
3	EHV-1	V592	AY464052.1	0.223173	0.167964	0	0.171514	0.134655	0.254362	0.271052	0.0924027	0.518862	0.27135	0.273263	0.270889	0.272349	0.204826	0.171923	0.26916	0.269123	0.519519
4	EHV-1	AB4	AY665713.1	0.225277	0.170318	0.171514	0	0.137453	0.255982	0.27301	0.0948288	0.519078	0.273444	0.275153	0.272982	0.27422	0.207164	0.170651	0.271444	0.271407	0.520018
5	**EHV-1**	**EHV-1/MA/2010/21**		0.189896	0.133556	0.134655	0.137453	0	0.161878	0.24024	0.0583345	0.495044	0.240065	0.241907	0.239604	0.240903	0.17236	0.131773	0.236644	0.234404	0.496044
6	**EHV-8**	**EHV-8/MA/2017/21**		0.21087	0.258709	0.254362	0.255982	0.161878	0	0.15793	0.179322	0.508757	0.147383	0.147581	0.147137	0.146946	0.28152	0.256082	0.141177	0.0934447	0.508588
7	EHV-8	Wh	JQ343919.1	0.234657	0.27544	0.271052	0.27301	0.24024	0.15793	0	0.196353	0.520573	0.15755	0.158938	0.15673	0.156215	0.301771	0.273191	0.171761	0.171761	0.520831
8	EHV-1	Hertfordshire/150/2016	KY852346.1	0.145958	0.0913324	0.0924027	0.0948288	0.0583345	0.179322	0.196353	0	0.467692	0.196451	0.198335	0.195993	0.197192	0.131515	0.0949388	0.194501	0.194335	0.468207
9	EHV-4	TH20p	LC063142.1	0.514498	0.524651	0.518862	0.519078	0.495044	0.508757	0.520573	0.467692	0	0.519015	0.519995	0.518787	0.51921	0.529935	0.519035	0.517322	0.516947	0.137573
10	EHV-8	EHV-8/IR/2003/19	MF431611.1	0.230274	0.275897	0.27135	0.273444	0.240065	0.147383	0.15755	0.196451	0.519015	0	0.145845	0.141	0.143735	0.298166	0.273858	0.157683	0.157683	0.519245
11	EHV-8	EHV-8/IR/2010/47	MF431612.1	0.231486	0.277591	0.273263	0.275153	0.241907	0.147581	0.158938	0.198335	0.519995	0.145845	0	0.145912	0.145829	0.299957	0.275567	0.16022	0.16022	0.5203
12	EHV-8	EHV-8/IR/2010/47	MF431613.1	0.229781	0.275433	0.270889	0.272982	0.239604	0.147137	0.15673	0.195993	0.518787	0.141	0.145912	0	0.143233	0.297761	0.273396	0.157591	0.157591	0.519017
13	EHV-8	EHV-8/IR/2015/40	MF431614.1	0.231148	0.276679	0.272349	0.27422	0.240903	0.146946	0.156215	0.197192	0.51921	0.143735	0.145829	0.143233	0	0.299105	0.274667	0.123034	0.123034	0.519398
14	EHV-1	KyA	MF975655.1	0.256233	0.202955	0.204826	0.207164	0.17236	0.28152	0.301771	0.131515	0.529935	0.298166	0.299957	0.297761	0.299105	0	0.206859	0.296621	0.296577	0.530822
15	EHV-8	YM2019	MT063054.1	0.225271	0.170127	0.171923	0.170651	0.131773	0.256082	0.273191	0.0949388	0.519035	0.273858	0.275567	0.273396	0.274667	0.206859	0	0.269159	0.269122	0.519982
16	EHV-8	SDLC66	MW816102.1	0.22769	0.273769	0.26916	0.271444	0.236644	0.141177	0.171761	0.194501	0.517322	0.157683	0.16022	0.157591	0.123034	0.296621	0.269159	0	0	0.517495
17	EHV-8	SD2020113	MW822570.1	0.227666	0.273739	0.269123	0.271407	0.234404	0.0934447	0.171761	0.194335	0.516947	0.157683	0.16022	0.157591	0.123034	0.296577	0.269122	0	0	0.517121
18	EHV-4	EHV4 génome complet	NC_001844.1	0.515115	0.525439	0.519519	0.520018	0.496044	0.508588	0.520831	0.468207	0.137573	0.519245	0.5203	0.519017	0.519398	0.530822	0.519982	0.517495	0.517121	0
